# Innovative role of Benzalkonium chloride as a quaternary ammonium salt for natural gas hydrate formation and storage

**DOI:** 10.1038/s41598-025-97520-3

**Published:** 2025-05-09

**Authors:** Mohamed S. Gad, Abeer M. Shoaib, Mustafa Awad, Hussien A. Elmawgoud, S. A. Khalil, A. M. Alsabagh

**Affiliations:** 1 Process Development Department, Egyptian Petroleum Research Institute (EPRI), Nasr City, Cairo, Egypt; 2 Applications Department, (EPRI), Nasr City, Cairo, Egypt; 3 Department of Petroleum. Refining and Petrochemical Engineering, Faculty of Petroleum and Mining Engineering, Suez University, Suez, 4351 Egypt

**Keywords:** Natural gas hydrate, Benzalkonium chloride (Bzc), Phase equilibrium, Induction time, Rate of hydrate formation, NG storage, Materials science, Energy science and technology, Natural gas, Energy, Chemical engineering, Chemistry, Process chemistry, Engineering, Chemical engineering

## Abstract

This study assessed the kinetics of natural gas hydrate (NGH) formation using N-benzyl-N, N-dimethyldodecan-1-aminium chloride (Benzalkonium chloride, Bzc) at concentrations ranging from 500 to 3000 ppm. It investigated its effects on the hydrate formation. These experiments were compared with pure water and sodium dodecyl sulfate (SDS) solutions at 298.15 K and 6.5 MPa. The findings indicate that the Bzc significantly enhances the formation kinetics and gas consumption of NGH. The 2500 ppm of Bzc notably reduced the induction time for hydrate nucleation up to 9.9 min. In contrast, it was 41.3 min with the SDS. The hydrate formation began at the gas/liquid interface and spread upward into the gas phase and downward into the liquid phase. The NGH dissociation and recovery were slower by the SDS among the Bzc solutions (smooth and fast). This observation indicates that the Bzc improves the formation and dissociation kinetics, making it a promising NGH formation and storage reagent. The results show that the Bzc significantly boosts the kinetics of NGH formation and dissociation at a small time and pressure. Providing valuable insights for optimizing hydrate technology.

## Introduction

Gas hydrates are solid, crystalline substances that form once water and gas molecules come together under elevated pressure and reduced temperature conditions. In these conditions, the water molecules create a lattice-like structure that traps the gas molecules within, forming what is known as a hydrate. These hydrates resemble ice but are unique due to their composition, where the gas molecules are encased within the water molecule framework^1,2^. In this arrangement, there is no chemical reaction (atom bonding) between the atoms of gas and water, allowing the gas molecules to orbit freely within the cages^3^. Relying on the size and nature of the guest molecules, gas hydrates can take on three different structures: structure I, II, and H. Much research has been dedicated to studying the phase equilibrium, thermodynamic stability, and kinetics of gas hydrate formation and dissociation^4,5^. Still, most of these studies focus on pure or binary gas mixtures^6–8^. Meanwhile, experimental research on hydrate formation and dissociation in gas mixtures composed of multiple components remains limited^9–12^. Natural gas composed of various hydrocarbons such as methane (CH_4_), ethane (C_2_H_6_), and propane (C_3_H_8_), along with non-hydrocarbon gases such as carbon dioxide (CO_2_), nitrogen (N_2_), and hydrogen sulfide (H_2_S), presents unique challenges because each component forms hydrates with different structures and equilibrium conditions. Understanding the influence of various factors on the properties of hydrate structure, dispersion of guest molecules, phase dynamics, and kinetics of NGH is essential due to their broad industrial applications. This includes natural gas storage and transport^13^, gas separation^14^, CO_2_ removal, CO_2_ capture and storage^15^, desalination of seawater^16^, and cryogenic storage. Natural gas storage as hydrates is particularly attractive because of its high gas storage capacity per unit volume, eco-friendly benefits^17,18^, and safety advantages. However, significant challenges like high pressure, low hydrate temperature, and slow growth formation rates persist^19^. To overcome these hurdles, various thermodynamic additives have been studied by numerous scientists, including solvents like tetrahydrofuran, acetone, and 1,4-dioxane, as well as quaternary ammonium compounds such as tetra-n-butyl ammonium bromide (TBAB) and tetra-n-butyl ammonium fluoride (TBAF) to lower the required pressure at equilibrium^20,21^. Quaternary Ammonium Salts (QAS) were selected due to their multifunctional role in enhancing natural gas hydrate (NGH) formation. QAS molecules effectively lower the interfacial tension between gas and water, increase the availability of gas molecules at the hydrate-water interface, and act as kinetic promoters by stabilizing hydrate nuclei during growth^22,23^. Their ability to inhibit competing reactions, such as ice formation at subzero temperatures, further makes them highly efficient in NGH systems. Moreover, QAS compounds are readily soluble in water and can be chemically tailored to optimize their performance for specific applications. Although toxicity concerns exist, these risks are minimized through precise concentration control and closed-system environments. Furthermore, their application is supported by emerging research on eco-friendly QAS derivatives and advanced methods for safe handling, ensuring their feasibility in hydrate storage and transportation technologies. Tetrahydrofuran (THF) has been extensively employed to decrease the pressure required for the formation of gas hydrates containing methane^24^, carbon dioxide^25^, methane-carbon dioxide mixtures, and carbon dioxide-nitrogen blends^26^. Research by Kang et al.^26,27^ investigated the three-phase equilibrium conditions of mixed hydrates comprising carbon dioxide, nitrogen, and tetrahydrofuran, finding that adding THF shifts the dissociation pressure to higher temperatures and lower pressures. Similarly, Zhong et al. found that in CO_2_-CH_4_ hydrates, THF adjusted the phase equilibrium conditions, favoring lower pressures. Lee et al. also explored the phase equilibrium behavior of methane-tetrahydrofuran, carbon dioxide-tetrahydrofuran, methane-carbon dioxide, and methane-carbon dioxide-tetrahydrofuran systems through a broad spectrum of temperatures, pressures, and concentrations, finding that THF consistently lowered the dissociation pressure and increased the temperature across various gas mixture compositions. They further observed that adding THF causes CH_4_ + CO_2_ hydrates to form structure II (sII). Additionally, Lee et al.^28^ investigated the stability and distribution of guest molecules within methane (CH_4_), ethane (C_2_H6), and propane (C_3_H_8_) hydrates in the presence of THF. They utilized thermodynamic and spectroscopic techniques to analyze how these components interact and stabilize under specific conditions^29^.

Surfactants like SDS are commonly used to speed up the formation of hydrate without altering the hydrate’s phase stability^30,31^. Despite their effectiveness, the exact process by which surfactants boost the hydrate formation rate remains a topic of ongoing discussion. Some research suggests that surfactants may form micelle-like structures in water, accelerating hydrate kinetics^32,33^. However, this theory is contested, with some studies arguing that micelles don’t form under hydrate-forming conditions^34^. The surface-active agents inhibit the creation of a thin, inflexible layer of hydrate at the boundary between the liquid and gas. Instead, these agents may hinder the formation of a compact hydrate layer at the interface, promoting the development of porous, thread-like crystals that enhance additional liquid absorption from the bulk phase due to the capillary action, as previously reported^15,35^. A few studies have also reported that surfactant adsorption on the surface of hydrate influences the kinetics of the growth of hydrate formations^36,37^. Therefore, a detailed molecular-level study is still needed to understand how surfactants fully promote hydrate formation.

This study aims to investigate the impact of a new surfactant, Bzc, on promoting natural gas storage in the form of hydrates. The potential of Bzc will be benchmarked against SDS and pure water systems in terms of the phase equilibrium conditions and formation kinetics of NGH. The work should be extended to determine the equilibrium conditions of NGH in Bzc and SDS in terms of induction time of nucleation stage, gas uptake, formation rate, water conversion, and storage capacity being evaluated to assess their impact on the kinetics of hydrate formation.

## Experimental

### Material measurements and methods

*Materials used natural gas*: the composition of natural gas was analyzed using gas chromatography (GC) equipped with a thermal conductivity detector (TCD) and a flame ionization detector (FID) to ensure accurate quantification of its components. A representative gas sample was collected in a gas-tight container and injected into the GC using a gas injection loop. The analysis was performed with helium as the carrier gas and appropriate chromatographic columns optimized for separating hydrocarbons and non-hydrocarbon gases. The sample components were separated based on their interactions with the stationary phase and detected as individual peaks. They were identified and quantified by comparing their retention times and peak areas against certified calibration standards. The method quantified significant components, including methane, ethane, propane, butanes, and pentanes, along with minor components, such as nitrogen, carbon dioxide, and hydrogen sulfide. Results were expressed in mole percent (mol%) and normalized to 100% for accuracy. Calibration and quality control adhered to ASTM D1945 and ISO 6974 standards to ensure the reliability of the measurements for energy content determination and quality assessment^38^. The composition of natural gas is shown in Table 1.

**Table 1:** Natural gas composition.

Composition	C_1_	C_2_	C_3_	IC_4_	NC_4_	IC_5_	NC_5_	NC_6_	NC_7_	NC_8_	NC_9_	N_2_	CO_2_
Mole %	95.447	2.550	0.654	0.178	0.163	0.082	0.059	0.059	0.056	0.022	0.002	0.066	0.662

**Fig. 1.**
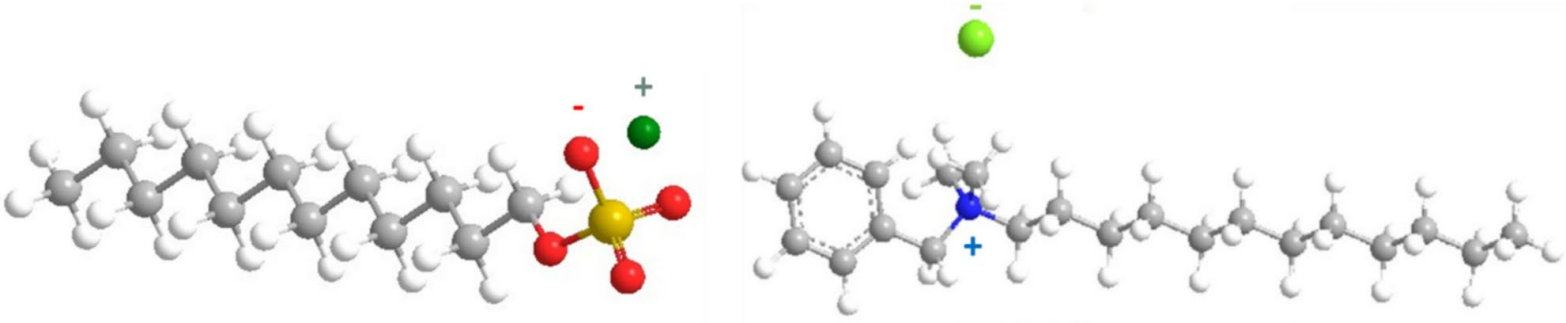
3D of chemical structure for sodium dodecyl sulphate (SDS) and Benzalkonium chloride (Bzc).

*Surfactant Used*: The anionic surfactant most used in gas hydrate formation, SDS, was used compared with the tested cationic surfactants. The used cationic surfactant is N-benzyl-N, N-dimethyldodecan-1-aminium chloride. The origin and purity of the surfactant are outlined in Table 2, and its chemical 3D structure is illustrated in Fig. 1.

**Table 2:** Materials used.

No	Material	Supplier	Purity, %
1	Natural gas mixture (methane)	Petrobel co. Aboumady Fields, Egypt	95.447
2	Benzalkonium chloride (Bzc)	Aldrich sigma	99.5
3	Sodium dodecyl sulfate (SDS)	Aldrich sigma	99.5
4	Distilled water	EPRI, Laboratory	4.9 × 10^–6^ (S m^–1^)

*Measurements, zeta potential measurement*: An accurate mass balance (RADWAG AS-220/X) was utilized to prepare the materials, ensuring measurements with a precision of ± 0.00004 mass fraction. The deionized distilled water of ‘Type 3’ grade was used to create the aqueous solutions. The surfactant solutions were analyzed with a Malvern Zetasizer Nano ZS equipped with a 633 nm laser. Samples were synthesized at 1 mg/mL concentration using 10 mM phosphate-buffered saline (PBS) at a pH of 7.4. Before measurement, the samples were sonicated for 5 min in a water bath Sonicator. The zeta potential was measured by laser diffraction electrophoresis at 25 °C^39^.

*Surface Tension Measurement*: The measurements were carried out using the Krüss K100 tensiometer equipped with a platinum ring. Bzc solution solutions are prepared at concentrations ranging from 200 to 3000 ppm, and SDS solutions ranging from 100 to 1000 ppm in deionized water. The results were performed at 6 °C^40^.

### NGH formation setup

**Fig. 2:**
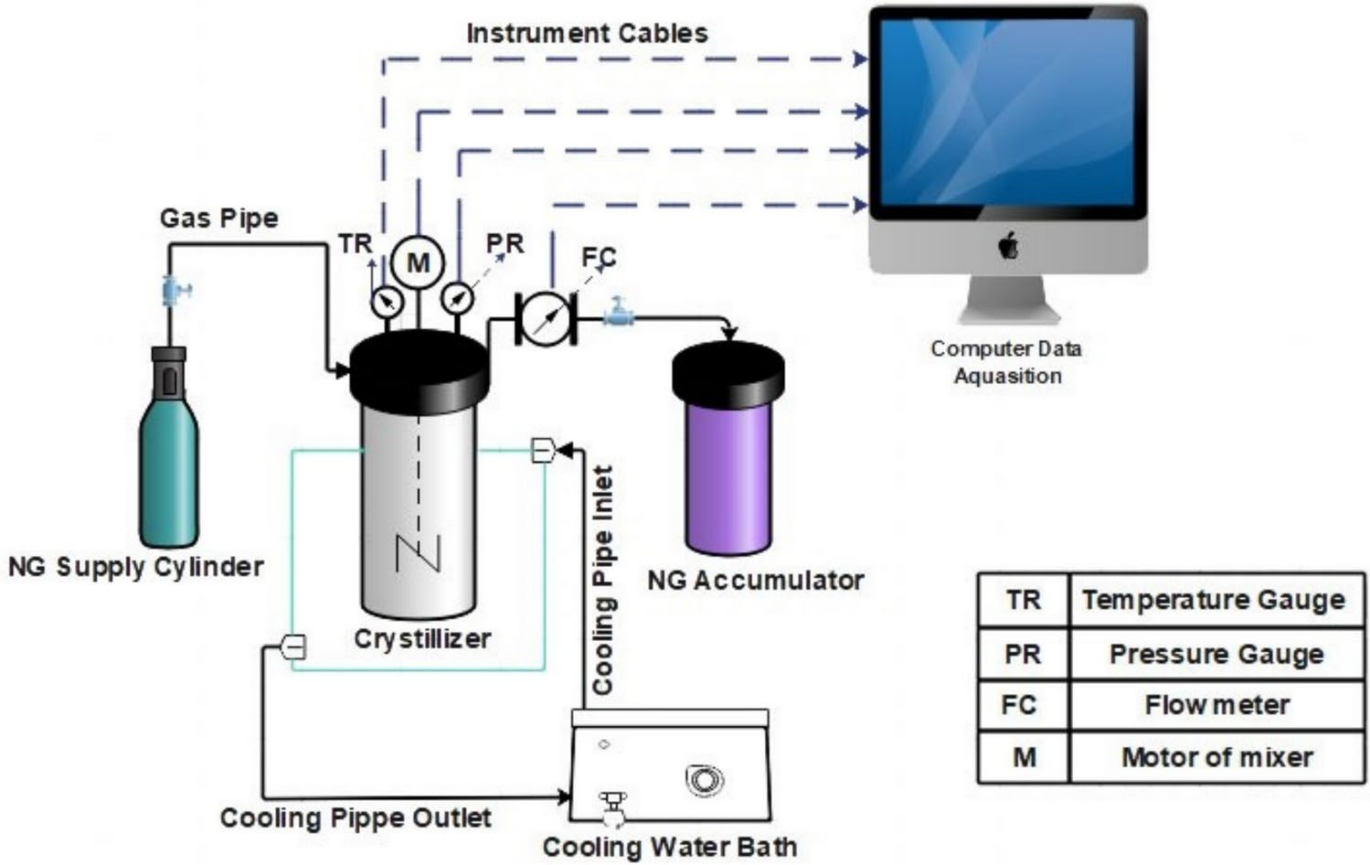
Diagram of the experimental apparatus setup.

The experimental setup for studying the kinetics of NGH formation in Bzc and SDS water-based mixtures is illustrated in Fig. 2. It includes a high-strength, corrosion-resistant crystallizer with a volume of 1.4 L. It is capable of withstanding pressures of up to 10 megapascals. This crystallizer has a temperature control system, a precision pressure gauge, and a high-accuracy thermometer (calibrated to ASTM 1137 standards). The crystallizer can operate at extremely high temperatures, reaching as high as 673 Kelvin. The pressure gauge (Model HD20V4T, Delta Ohm) provides precise readings of the internal reactor pressure, with a margin of error of ± 0.02 megapascals, while the thermometer has an uncertainty of ± 0.11 Kelvin.". In a temperature-control water bath, the flowing glycol–water mixture circulates through the jacket to achieve the targeted thermal conditions, regulated by a PID controller. A magnetic stirrer inside the crystallizer ensures proper mixing, while data from the sensors are continuously recorded and transmitted to a computer system for storage via a control panel^41^.

### Procedure of NGH formation

The study involved two main types of experiments: hydrate stability zone (HSZ) tests and hydrate kinetics studies. The HSZ tests aimed to determine the pressure and temperature conditions under which the hydrate phase would be stable. The experimental setup began by filling a 600 cm^3^ crystallizer cell with pure water. In some cases, the water was doped with the promoter Bzc at varying concentrations to observe its effects. The air above the liquid was evacuated, and the desired gas was charged into the crystallizer cell until the target pressure was reached. The degrees of temperature within the cell were carefully lowered in a series of incremental steps, beginning at an initial temperature of 294.15 K, using a precision temperature control device. At each stage, the system was given a 60-min window to settle and attain a state of equilibrium. The formation of hydrates was subsequently tracked, marked by a sudden and pronounced decrease in pressure. Once hydrate formation was confirmed, the temperature was further reduced in small, 3–4 °C increments to chart the complete hydrate stability zone thoroughly. The initial pressure and temperature conditions were set outside the hydrate stability region in the hydrate kinetics studies. The temperature was then lowered to trigger the nucleation of hydrates, which was observed as a rise in temperature and a pressure drop^42^. The induction time (time to nucleation) and the pressure–temperature evolution over time were recorded. Each kinetics experiment was repeated three times to verify the results, which showed pressure variations within ± 0.1 MPa. After the hydrate formation experiments, the behavior of dissociation of the generated hydrates was investigated using a method of thermal stimulation, as described in^43^. The dissociation experiment protocol was as follows: the temperature of the air bath surrounding the experimental system was increased from the final hydrate formation temperature up to 298.15 K. This temperature ramp was applied at 0.15 K/min. In contrast, the system was maintained under static (closed) conditions. As the temperature increased, the pressure within the system initially rose gradually. This was due to the thermal expansion of the gas phase. However, the solid hydrate phase dissociated once the experimental temperature reached the equilibrium value corresponding to the prevailing pressure. The dissociation of the hydrate structure caused a rapid increase in pressure as the natural gas previously captured within the hydrate was released. The pressure and temperature remained constant, indicating the completion of the hydrate dissociation process. The final pressure value was slightly higher than the initial pressure before dissociation. This discrepancy was attributed to the thermal expansion of the released gas. By employing this thermal stimulation approach, the complete dissociation behavior of the formed hydrates was observed and documented. The collected pressure and temperature data provided valuable insights into the dissociation characteristics of the hydrates under the tested conditions.

### Calculation method

The data (pressure–temperature) recorded throughout the trials determined the quantity of natural gas absorbed (consumed) in the formation of hydrate, the rate at which the hydrate formed, the proportion of gas converted to hydrate, and the efficiency of natural gas release or recovery.

#### The number of moles of gas consumption

The number of gas moles consumed in forming hydrate or semi-clathrate hydrate was calculated using Eq. (1).

1 $$\Delta {\text{n}}_{{ \downarrow ,{\text{H}}}} = {\text{V}}_{{\text{g}}} \left( {\frac{{{\text{P}}^{{\text{i}}} }}{{{\text{Z}}^{{\text{i}}} {\text{RT}}}} - \frac{{{\text{P}}^{{\text{t}}} }}{{{\text{Z}}^{t} {\text{RT}}}}} \right)$$

This equation involves several key variables: the amount of natural gas used up at a given time (Δn↓,H), the volume of gas inside the crystallizer (Vg), the initial pressure when hydrate formation began (Pi), the initial compressibility factor (Zi), the ideal gas constant (R), the average temperature of the gas throughout the experiment (T), the pressure inside the reactor at a given time (Pt), and the compressibility factor at that time (Zt). The values of the initial and instantaneous compressibility factors are derived using the Pitzer correlation method, as outlined by Smith et al. in 2001. The volume of natural gas consumption per unit volume of water is then calculated by using a separate Eq. (2).

2 $${\text{N}}_{{\text{t}}} = \Delta {\text{n}}_{ \downarrow } \left( {{\text{mole}}/{\text{mole}}} \right) = \Delta {\text{n}}_{{ \downarrow ,{\text{H}}}} /{\text{n}}_{{\text{w}}}$$

#### Gas-to-hydrate conversion

The percentage of conversion of Gas-to-hydrate (GH) at the end of each trial is determined by Eq. (3)^7^:

3 $${\text{GN}} = \Delta {\text{n}}_{{ \downarrow, {\text{ H}}}} /{\text{n}}_{{\text{w}}} * \frac{100}{\text{n}^{\rm g}}$$

where, n_g_ is the number of moles of natural gas in the crystallizer at the beginning of the trial.

#### The rate of hydrate formation

The rate at which NGH formed was calculated using a step-by-step numerical approach, which involved examining the changes in the system at regular intervals, as outlined in Eq. (4)^44^.

4 $$\frac{dn}{{ dt }} = \left( {\frac{{d\Delta_{n \downarrow ,H} }}{dt }} \right)_{t} = \Delta_{n \downarrow ,H} \frac{{\left( {\Delta_{n \downarrow ,H,t} + \Delta t - \Delta_{n \downarrow ,H t} } \right)}}{\Delta t }$$

Here, Δt represents the time interval between two measurements, set at 30 s, and the average rate of NGH formation was determined at hourly intervals and graphically illustrated. The efficiency of NG recovery has been calculated using Eq. (5)^45^:

5 $${\text{NG}}\;{\text{recovery}} = \Delta {\text{n}}_{{ \downarrow ,{\text{H}}}} /{\text{n}}_{{\text{g}}} *{1}00$$

## Results and discussion

**Fig. 3:**
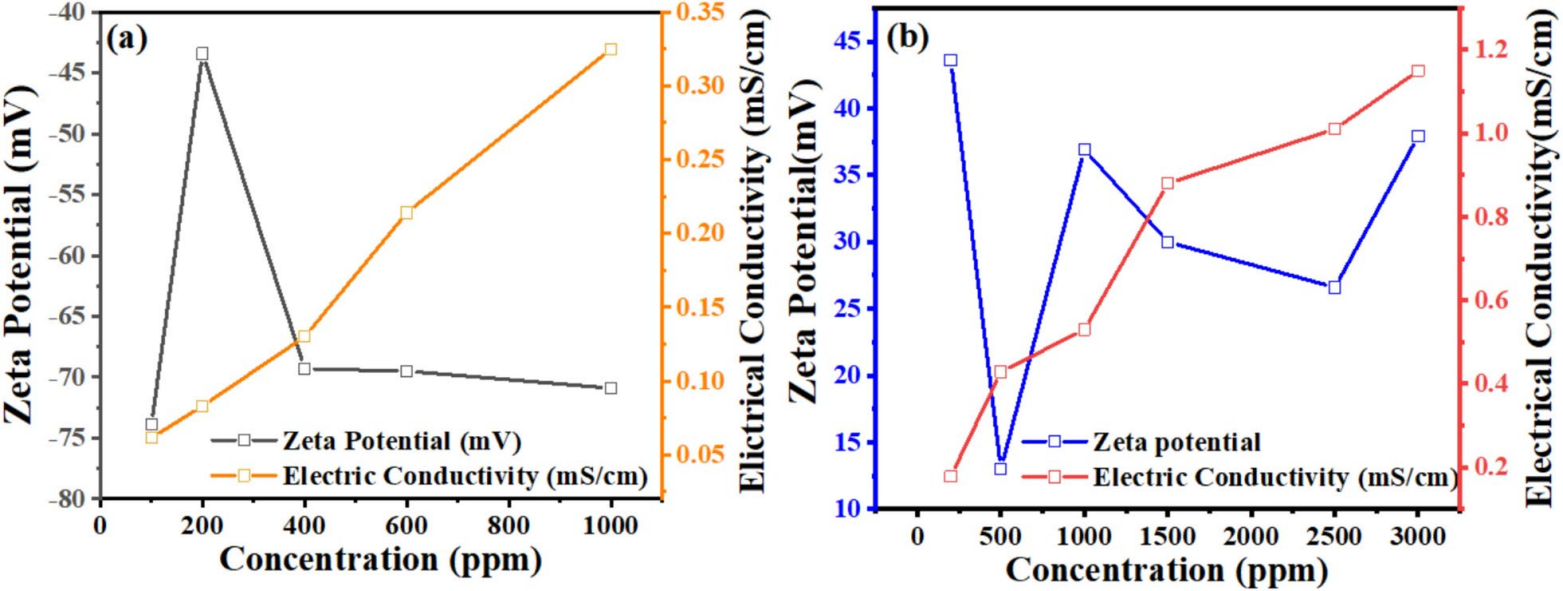
Electrical conductivity and zeta potential in **a** Different Concentration of SDS from 100 ppm to 1000 and **b** Different Concentration of Bzc from 200 to 3000 ppm.

The importance of natural gas in our modern era is significant, as many contemporary approaches focus on storing natural gas in the form of hydrates. Various methods exist for this purpose, but this work specifically concentrates on using cationic surfactants to create gas hydrates. It compares their performance with the well-known SDS commonly used in hydrate formation and storage. In this context, the study also emphasizes analyzing the cationic surfactant solutions used, mainly focusing on the zeta potential and surface tension measurements to determine their role in hydrate formation. Zeta potential was measured to follow the stability of the electrical double layer of surfactant solutions. Zeta potential provides insights into the stability and charge distribution of surfactant molecules in solution, which affects micelle formation^46^. The surface tension measurements indicate the point at which surfactant molecules begin to aggregate and form micelles, marking the CMC. Together, these measurements offer a comprehensive understanding of surfactant behavior and are crucial for optimizing surfactant concentrations in the hydrate formations. In Fig. 3 and Table 3, the presented data demonstrates the relationship between conductivity and zeta potential measurements used to determine the critical micelle concentration (CMC) of SDS and Bzc surfactants.

**Table 3:** The results of zeta potential and conductivity for different concentrations of Bzc and SDS.

Sample	Weight (g/l)	Concentration (ppm)	Zeta potential (mV)	Conductivity (mS/cm)
Bzc	0.2	200	43.6	0.178
Bzc	0.5	500	13	0.429
Bzc	1	1000	36.9	0.53
Bzc	1.5	1500	30	0.88
Bzc	2.5	2500	26.6	1.01
Bzc	3	3000	37.9	1.15
SDS	0.1	100	− 73.9	0.0614
SDS	0.2	200	− 43.4	0.083
SDS	0.4	400	− 69.3	0.13
SDS	0.6	600	− 69.5	0.214
SDS	1	1000	− 70.9	0.325

The conductivity curve for SDS exhibits a clear inflection point around 600 ppm, indicating the CMC, where the linear increase in conductivity below the CMC transitions to a slower increase as micelles form. Similarly, the Bzc conductivity curve shows that the relationship between electrical conductivity and surfactant concentration is a direct proportional.

**Fig. 4:**
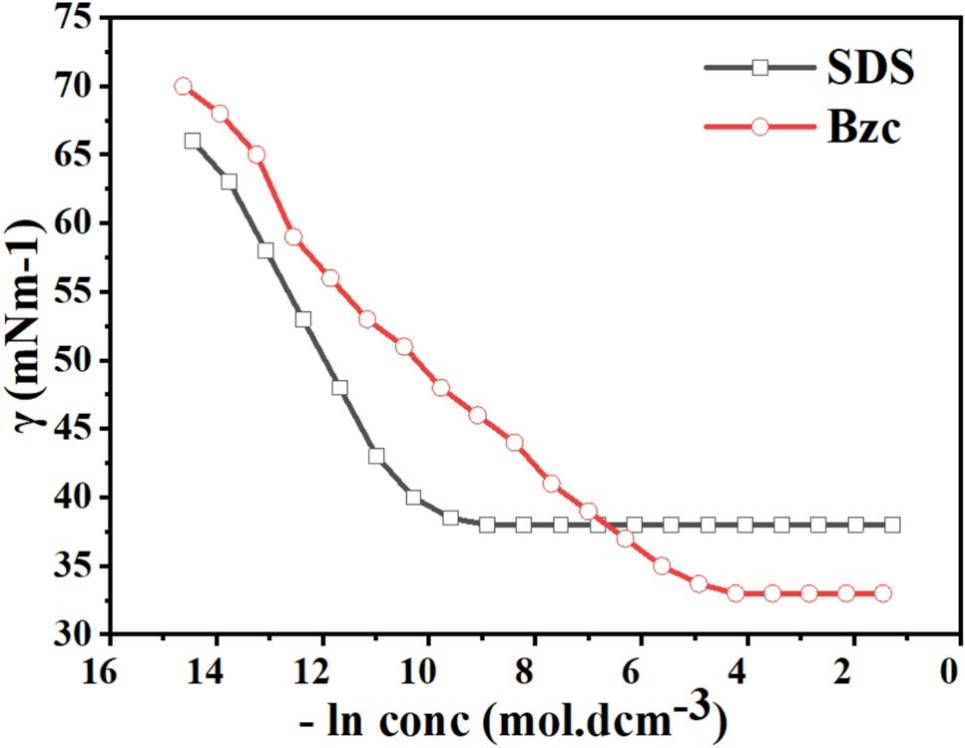
γ -lnC isotherm for the Used Surfactant at 6 °C.

**Fig. 5:**
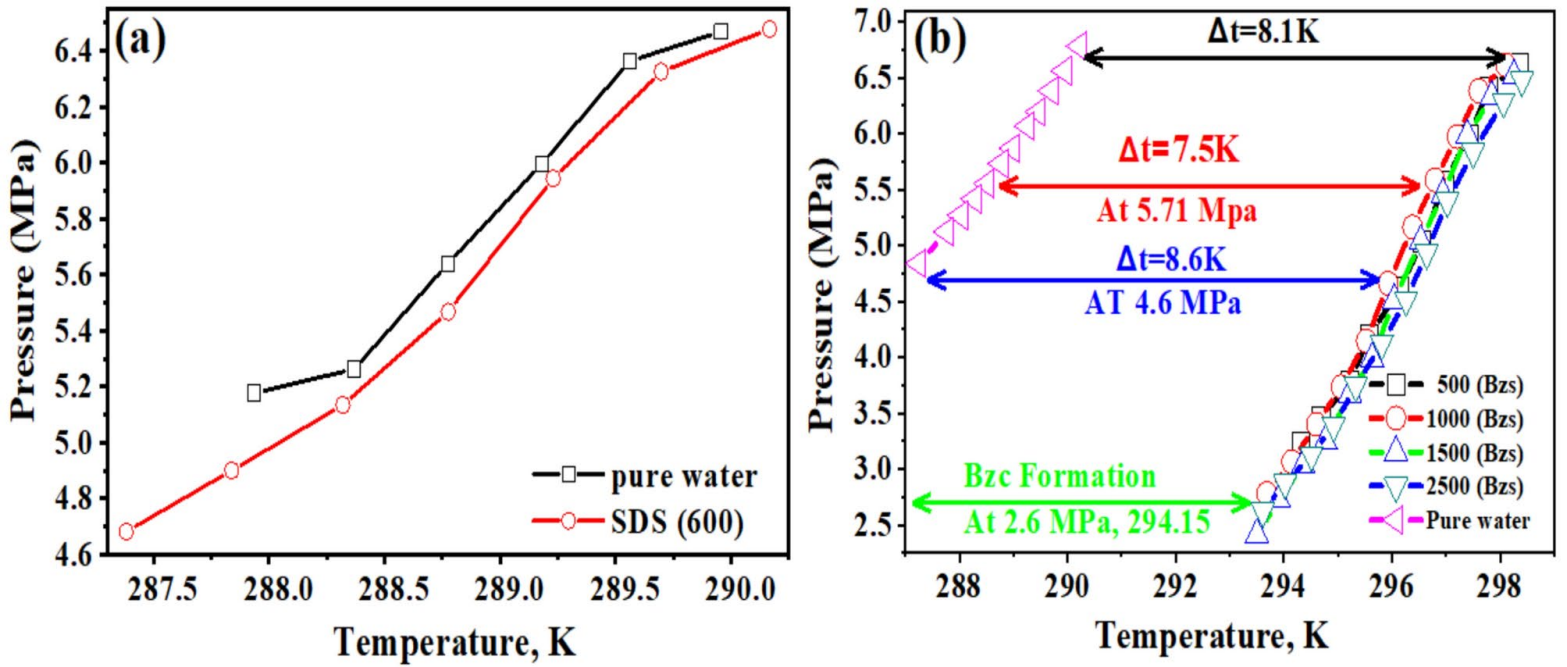
Phase equilibrium of natural gas hydrates in the presence of **a** Pure Water—SDS Concentration 600 ppm **b** Different Bzc concentrations.

The zeta potential curves for both surfactants undergo distinct changes at their respective CMC concentrations, further confirming the onset of micelle formation. Moreover, both surfactants show a decrease in surface tension with rising concentration until reaching a plateau, as illustrated in Fig. 4, indicating the critical micelle concentration (CMC). The Bzc reaches its CMC earlier and at (37 Mol.dcm^−3^) surface tension compared to Bzc (34 Mol.dcm^-3)^ at 6 °C, which implies that Bzc is more efficient at lowering surface tension. However, Bzc continues to lower surface tension beyond SDS levels, suggesting different micelle formation dynamics or molecular interactions in the solution. These observations provide valuable insights into the self-assembly behavior of these surfactants, enhancing the hydrate formation. Various aqueous solutions: pure water, pure water supplemented with one of the concentrations of SDS (600 ppm)^47^, and pure water supplemented with different concentrations of Bzc (2500, 1500, 1000, and 500 ppm) were studied in the term of phase equilibrium, induction time, the rate of hydrate formation and NG recovery. Bzc is a thermodynamic promoter that reduces the temperature or pressure necessary for hydrate creation. By incorporating Bzc at different concentrations (parts per million), the phase equilibrium conditions of the NGH system can be modified, leading to an increased storage capacity. This allows for more efficient utilization of the storage medium. Bzc facilitates the formation of NGH by providing suitable nucleation sites and reducing the energy barrier for hydrate crystal growth. This property enables the rapid and controlled formation of NGH during the storage process, enhancing overall storage efficiency. Using Bzc as a thermodynamic promoter allows for reversible hydrate formation and dissociation. This means the natural gas can be easily stored in the hydrate form and released when needed, providing a flexible and controllable storage option. These experiments aimed to understand how these additives affect the formation and dissociation of NGH. Hydrate formation was first initiated in pure water, and subsequent trials were performed in Bzc surfactant solutions at concentrations ranging from 500 to 2500 ppm and in a solution containing 600 ppm of SDS. This comparison allowed for finding changes in formation and dissociation conditions compared to the pure water system. A representative profile showing the relation between pressure and temperature during the hydrate formation in a 600 ppm SDS solution and Bzc (500, 1000, 1500, 2500 ppm) is depicted in Fig. 5a,b. Before nucleation, a slight pressure drop occurs as gases dissolve into the aqueous solution. Upon nucleation, a significant decrease in pressure occurs as the gas becomes encapsulated within the molecular frameworks created by water.

### The phase equilibrium of NGH formations

The experiments were carried out under specific conditions: an initial pressure of 6.5 MPa and a temperature of 273.15 K. Details of these experiments can be found in Table 4. The duration of the experiments was 24 h from the start of hydrate formation. The tests focused on the NGH system’s kinetics, specifically investigating the effects of two promoters, SDS and Bzc. To ensure comparable promotional effects on the pure hydrate system’s phase equilibrium, the SDS and Bzc concentrations were carefully selected. Figure 5a,b illustrate the phase equilibrium data for the pure hydrate/semi-clathrate hydrate system in the presence of SDS and Bzc at different volumes.

**Table 4:** Induction time, final gas consumption, gas conversion to hydrate, and NG recovery during hydrate formation at different concentrations of Bzc and SDS (600 PPM).

Aqueous system	EXP. No	Additives Conc., ppm	Induction time, Min	No. of mol gas/mol water	Gas to hydrate conversion, %	NG recovery, % (Dissociation)
NG + pure water	1	–	49	0.0391	42.02	–
	2		48.1	0.0382	41.06	–
	3		49.9	0.04013	43.13	–
	Ave		49	0.0391	42.02	–
NG + SDS	1	600	41	0.04201	45.15	95.40
	2		41.1	0.04165	44.77	95.35
	3		42	0.04289	46.10	95.41
	Ave		41.3	0.042	45.14	95.38
NG + Bzc	1	500	38	0.01715	18.43	95.60
	2		37.5	0.01724	18.53	95.54
	3		37	0.01608	17.30	95.58
	Ave		37.5	0.01720	18.49	95.57
NG + Bzc	1	1000	32	0.02786	29.95	95.80
	2		32.4	0.02811	30.11	95.79
	3		33	0.02801	30.09	95.81
	Ave		32.4	0.02799	30.08	95.80
NG + Bzc	1	1500	22	0.03278	35.24	96.55
	2		22.1	0.03108	33.41	96.67
	3		21.9	0.03256	34.99	96.80
	Ave		22	0.03214	35.54	96.67
NG + Bzc	1	2500	10	0.04155	44.66	98.30
	2		9.8	0.04132	44.42	98.58
	3		9.7	0.04142	44.52	98.80
	Ave		9.9	0.04143	44.50	98.56

The variable ΔT represents the boosting effect, calculated by finding the temperature difference between the equilibrium points of a pure hydrate or semi-clathrate hydrate system containing either SDS or Bzc, and the equilibrium point of a pure hydrate system at the same pressure. Comparing the phase equilibrium pressures, the NGH formed in pure water at 289.2 K has a pressure of 5.71 MPa, while the pressure for hydrate formed in the presence of SDS at 289.15 K is slightly higher at 5.74 MPa. This suggests that incorporating SDS does not notably change the phase equilibrium conditions. Other investigators have reached similar findings by examining different systems. The three-phase equilibrium conditions were investigated in the gas mixture containing CH_4_, C2H_6_, C_3_H_8_, hydrate, and SDS in an aqueous solution^48,49^. They conducted experiments at four different temperature points (275.2 K, 277.0 K, 278.8 K, and 280.6 K) using an isochoric isothermal dissociation technique and found that SDS does not affect the phase equilibrium. Verrett et al.^26^ demonstrated that while SDS does not change the bulk solubility of methane at concentrations that support hydrate growth, it does increase the methane concentration in the bulk liquid after nucleation. This indicates that the presence of SDS doesn’t alter the stability of hydrate formation conditions but does enhance the growth rate. Similarly, Gayet et al.^50,51^ noted that SDS does not affect the methane hydrate equilibrium compared to pure water systems. In summary, the cited research indicates that the introduction of SDS does not significantly impact the phase equilibrium of NGH. Instead, SDS enhances the growth rate without altering the equilibrium conditions. Including Bzc in the water system results in a notable increase in the equilibrium temperature. Specifically, at a pressure of 4.6 MPa, the equilibrium temperature rises to 295.9 K. This corresponds to an approximate temperature increase of 7.8 K under the same conditions. This finding suggests that the presence of Bzc causes a shift in the phase equilibrium curve of NGH in an aqueous solution, leading to a decrease in pressure and an increase in the equilibrium temperature (Δt = 8.6 K). Bzc is an organic compound that is heterocyclic and soluble in water. It readily forms structure II (sII), which hydrates with water in the form (51,264) of crystal structure, as it looks like the TBAB compound. From these observations, it can be concluded that the Bzc acts as an effective thermodynamic promoter. Its presence facilitates the formation of hydrates at lower pressures, which may be beneficial for storing natural gas in hydrate form. This is to be demonstrated in Fig. 5b.

### NGH induction time

**Fig. 6:**
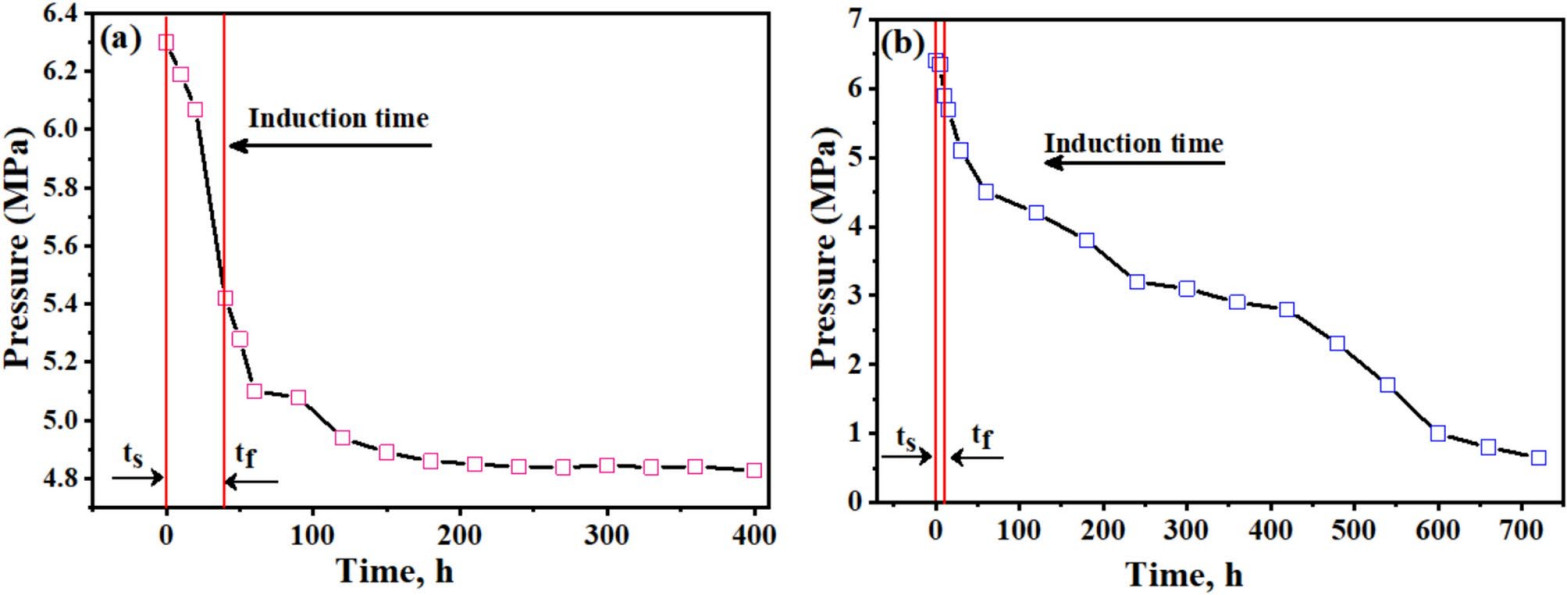
Time–Pressure response during hydrate formation of **a** 600 ppm SDS, **b** 2500 ppm Bzc Concentration.

In the context of gas hydrate formation, induction time refers to the delay between the initiation of the experimental conditions (e.g., pressure, temperature) and the onset of hydrate nucleation and growth. The induction time is an essential parameter in understanding the kinetics of hydrate formation, as it represents the initial period required for the system to reach the necessary supersaturation and form the first hydrate crystals^13^. The induction time can be influenced by various factors, such as the driving force for hydrate formation (pressure and temperature conditions), the presence of promoters or inhibitors, the mixing and agitation of the system, and the availability of nucleation sites. Measuring and analyzing the induction time can provide insights into the mechanisms and kinetics of hydrate formation, which is crucial for understanding and optimizing gas hydrate processes ^2^. Induction time refers to the period before a noticeable quantity of hydrate phase becomes apparent or until a measurable amount of hydrate-forming gas is consumed ^1^. Figure 6a,b show the variations in pressure and temperature over time through the hydrate formation in 600 ppm SDS solution with varying Bzc concentrations as the temperature drops by up to 6.7 K. The induction time, defined as the period between the start of the experiment (t_s_) and the onset of hydrate formation (t_f_), is reduced when SDS is added compared to pure water, where the induction time is recorded at 49 min. For instance, with 600 ppm SDS in water, the induction time is reduced to 41.3 min.

**Fig. 7:**
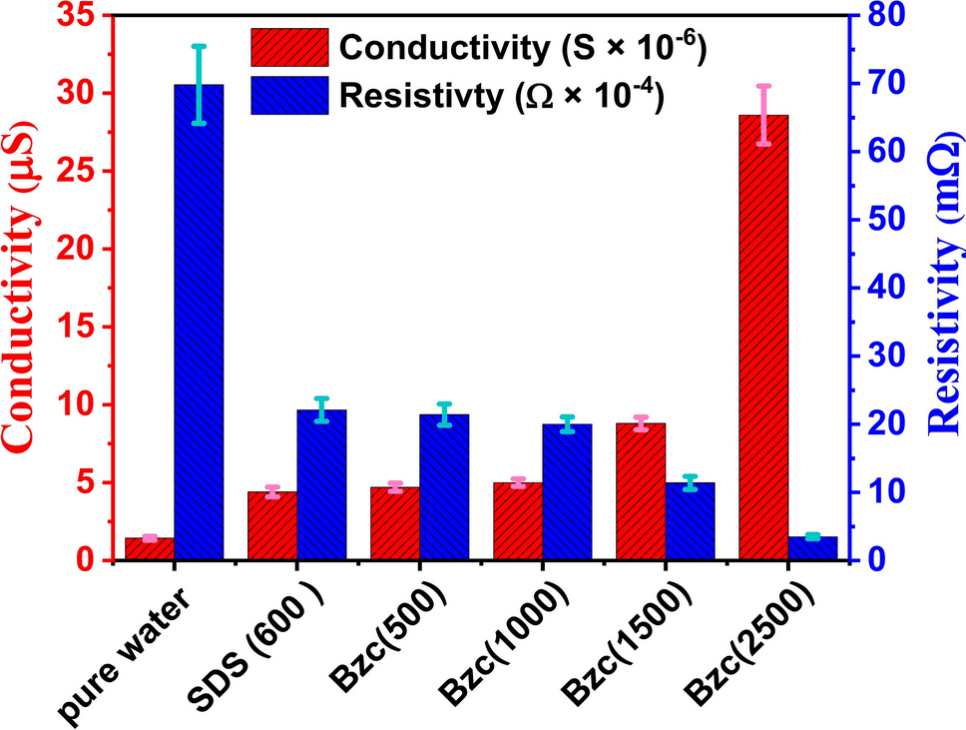
Conductivity and resistivity at different concentrations of Bzc and SDS (600 ppm) and pure water.

As the SDS concentration in water increases, the induction time decreases further. As mentioned before, this reduction in time due to SDS has also been documented^52,53^. With the supplement of Bzc to the water system, the induction time is further reduced by increasing its concentration. As shown in Table 4, the induction time was 37.5, 3.4, and 9.9 min against concentrations of 500, 1000, 1500, and 2500 ppm, respectively. Regarding the effect of electrical conductivity on the formation time of gas hydrates and the equilibrium state of the hydrates, high electrical conductivity facilitates faster transfer of charges and ions within the material. Furthermore, leads to an increase in the formation rate of NGHs. Materials with high electrical conductivity, such as salts and metals, help to speed up the formation of NGH compared with low-conductivity materials^15,46^. Table 3 shows the data obtained from measuring electrical conductivity and zeta potential at concertation of Bzc and comparing it with pure water and SDS. The measured conductivity and resistivity of different dosages of Bzc, SDS (600 ppm), and pure water were illustrated in Fig. 7. To ensure data reliability, the resistivity and inverse of every prepared solution were measured five times. The error values, resistivity (light blue), and conductivity (purple) were inserted as error bars in the figure. The data indicates that the maximum error reached 9% for the resistivity of pure water, while the minimum error was approximately 3% for the Bzc solution at a concentration of 2500 ppm. Otherwise, Electrical conductivity affects the strength of the hydrogen bonds in the crystal structure of NGH. tend to stabilize the crystal structure of the hydrates and increase their stability^10,54,55^. This stabilization of hydrate structure is attributed to these materials can enhance ionic mobility, promote favorable electrostatic interactions, improve thermal management, improve heat transfer, and stabilize phase behavior. Moreover, conductive materials can influence the hydrogen bonds within the gas hydrate lattice by modifying the electronic environment within and around the hydrate structure. These materials can enhance the alignment and distribution of water molecular dipoles, which strengthens dipole–dipole interactions critical to maintaining the integrity of the hydrate lattice. Additionally, the presence of these materials can lead to localized polarization effects and increase the strength of van der Waals forces. By stabilizing these intermolecular forces, conductive materials help to reinforce the crystal lattice, reducing the likelihood of structural disruptions and promoting a more robust and stable hydrate framework under varying environmental conditions. These factors collectively contribute to the overall stability and the reduction of induction time hydrate formation^10,56^.

This leads to an expansion of the thermal stability region of NGH at certain temperatures and pressures. Therefore, increased electrical conductivity helps to stabilize the state of NGH and broaden the equilibrium conditions specific to them^57,58^.

**Fig. 8:**
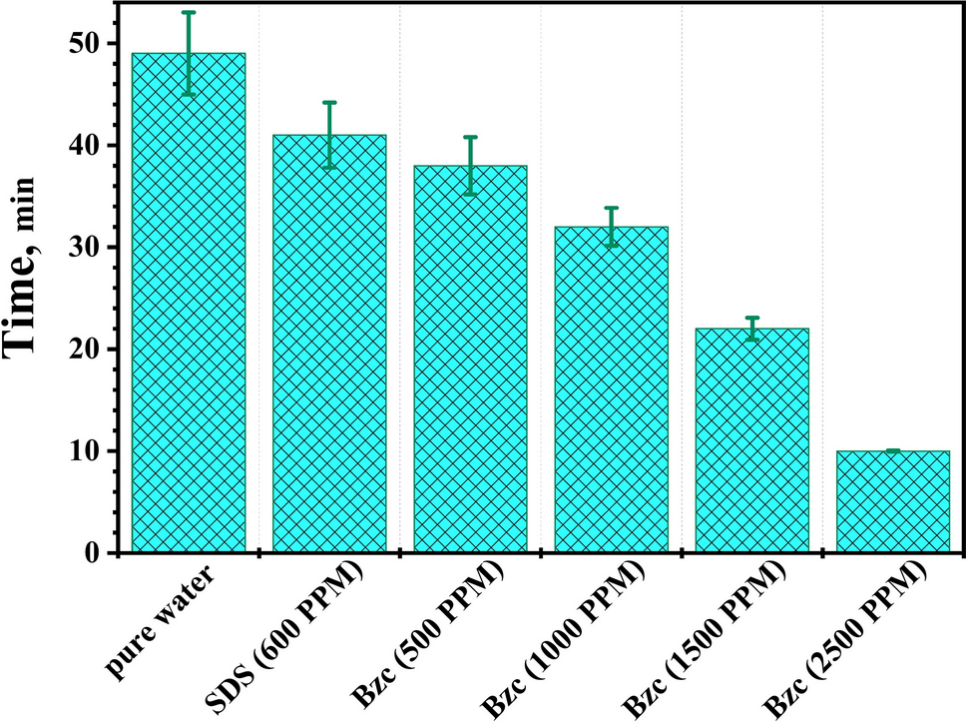
Variation of induction time at different concentrations of Bzc and SDS aqueous solution.

Figure 8 illustrates how introducing varying amounts of Bzc and SDS at a concentration of 600 ppm affects the induction time. Based on the obtained data, the error bars show that the maximum value is 8.6% for pure water, while the minimum value is 5.4% for Bzc (2500 ppm). This comparison reveals that one promoter is more effective at improving the induction time than the other.

### NGH formation among Bzc and SDS

**Fig. 9:**
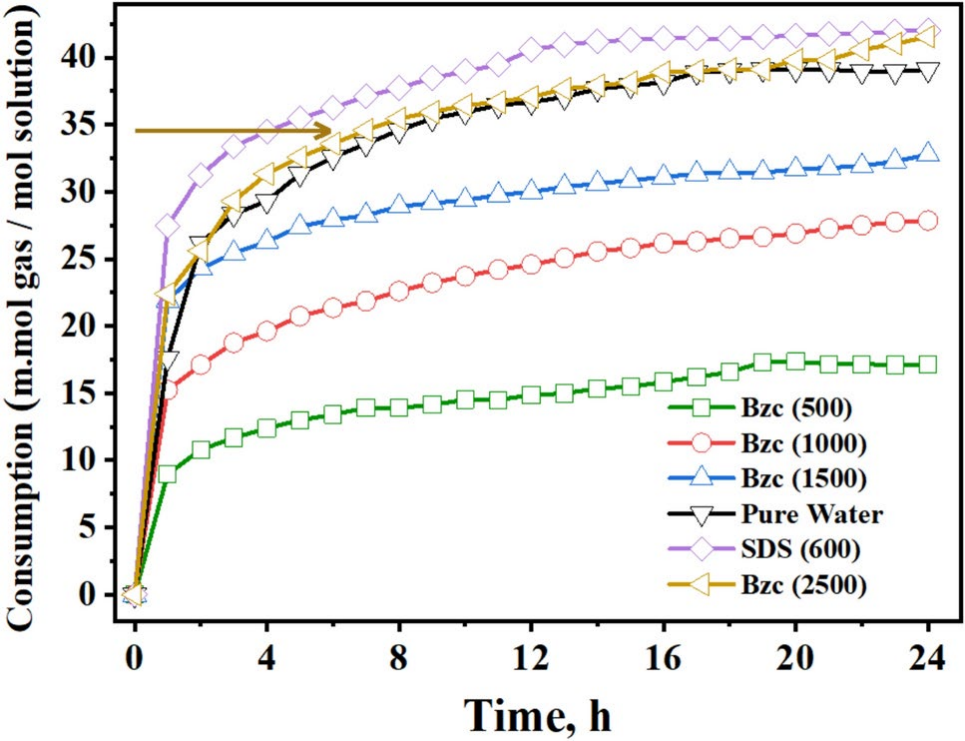
Gas consumption/mole water during hydrate formation at different concentrations of Bzc, SDS, and Pure Water.

Research has been conducted on the kinetics of the NGH formation using Bzc aqueous solutions at various concentrations (500, 1000, 1500, and 2500 ppm), as shown in Fig. 9. This figure shows the amount of the gas consumed per mole of water through (24-h) the period of hydrate formation, with t = 0 marking the onset. Notably, the 2500 ppm Bzc solution demonstrated the highest gas consumption among the other concentrations.

By increasing the concentration up to 2500, the number of mole gas consumed in the hydrate was being to 0.04211 mol; otherwise, at the concentration of SDS at 600 ppm, the number of moles was 0.4255 mol as reported before. This means that the Bzc exhibited a close number of gas consumption in the hydrate formation and with the SDS. During hydrate formation, the gas consumption curve levels off after a few hours, indicating no further hydrate formation. This rise in gas consumption is due to Bzc or SDS supplements.

**Fig. 10:**
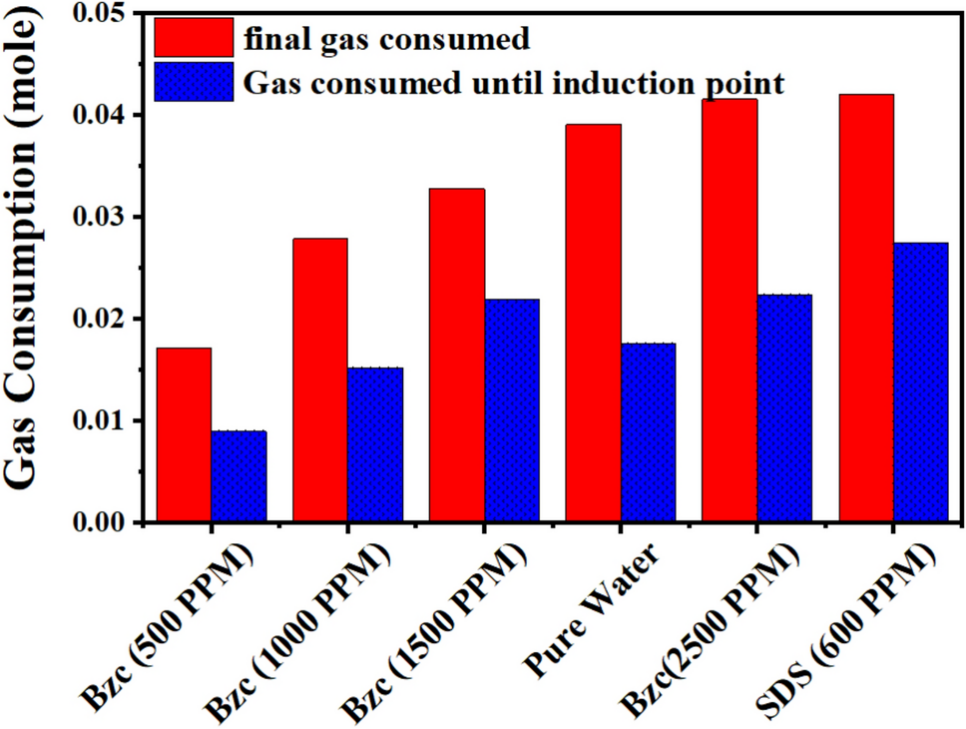
Comparative plot between gas uptake until induction point and final gas uptake.

**Fig. 11:**
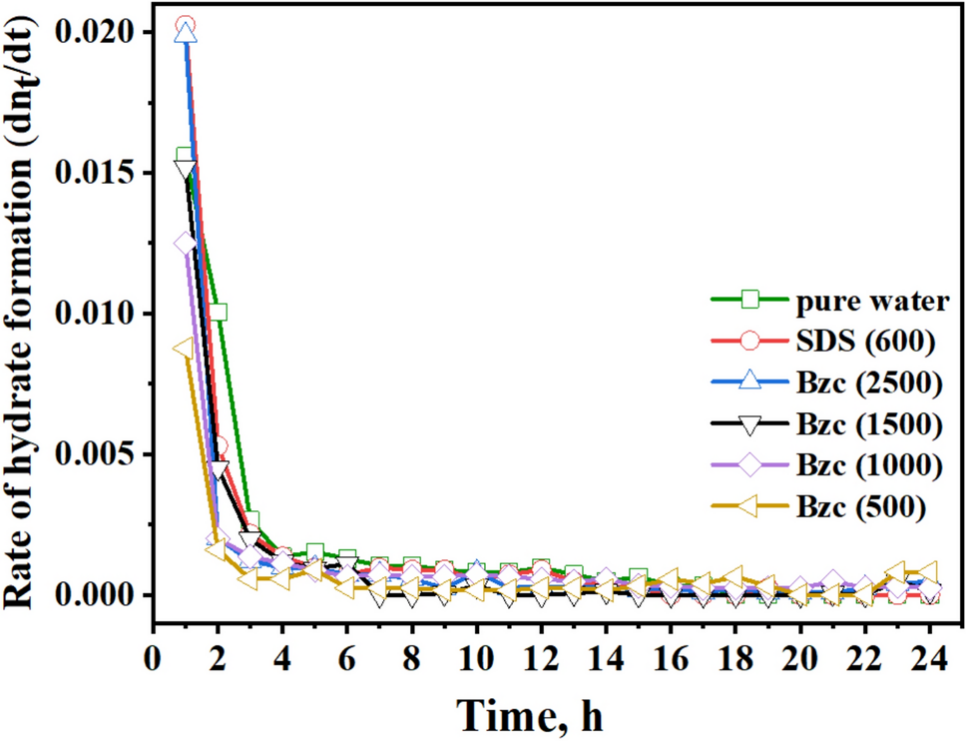
Rate of hydrate formation (mol/mol) vs. time of (pure water, SDS at different concentrations of Bzc, t1/4 24 h).

Figure 10 compares the gas consumed from the nucleation period to the final gas uptake. By analyzing the data, one can conclude that the minimum gas consumption until the nucleation, after which it increases rapidly, as reported elsewhere^26^. One notable benefit of utilizing Bzc is its ability to decrease the induction time relative to pure water and SDS by facilitating faster nucleation (refer to Table 4). Figure 11 illustrates the rate of NGH formation at various concentrations of Bzc aqueous solution, alongside a comparison to pure water and pure water containing SDS (600 ppm) aqueous solution.

In the early stages of the hydrate formation experiment, spanning the first three hours, the SDS-based system demonstrated a rapid hydrate growth rate, followed by Bzc (2500 ppm), pure water, Bzc (1500 ppm). The hydrate formation rate was tested with Bzc at 1000 ppm and then at 500 ppm. However, the rates for Bzc at 2500 ppm, pure water, and pure water with SDS were nearly identical, gradually slowing to zero by the final of the (24-h) hydrate formation test. When comparing the rate of NGH formation among Bzc, SDS, and other systems, it was observed that the systems with SDS at 600 ppm and Bzc at 2500 ppm showed the highest amount of gas consumption per mole of water in the NGH system opposite to other concentrations. Overall, using Bzc in an aqueous solution appears to enhance gas consumption. These findings indicate that further investigation is needed to fully understand the role of surfactants and various promoters in hydrate formation.

### NG release through dissociation stage

**Fig. 12:**
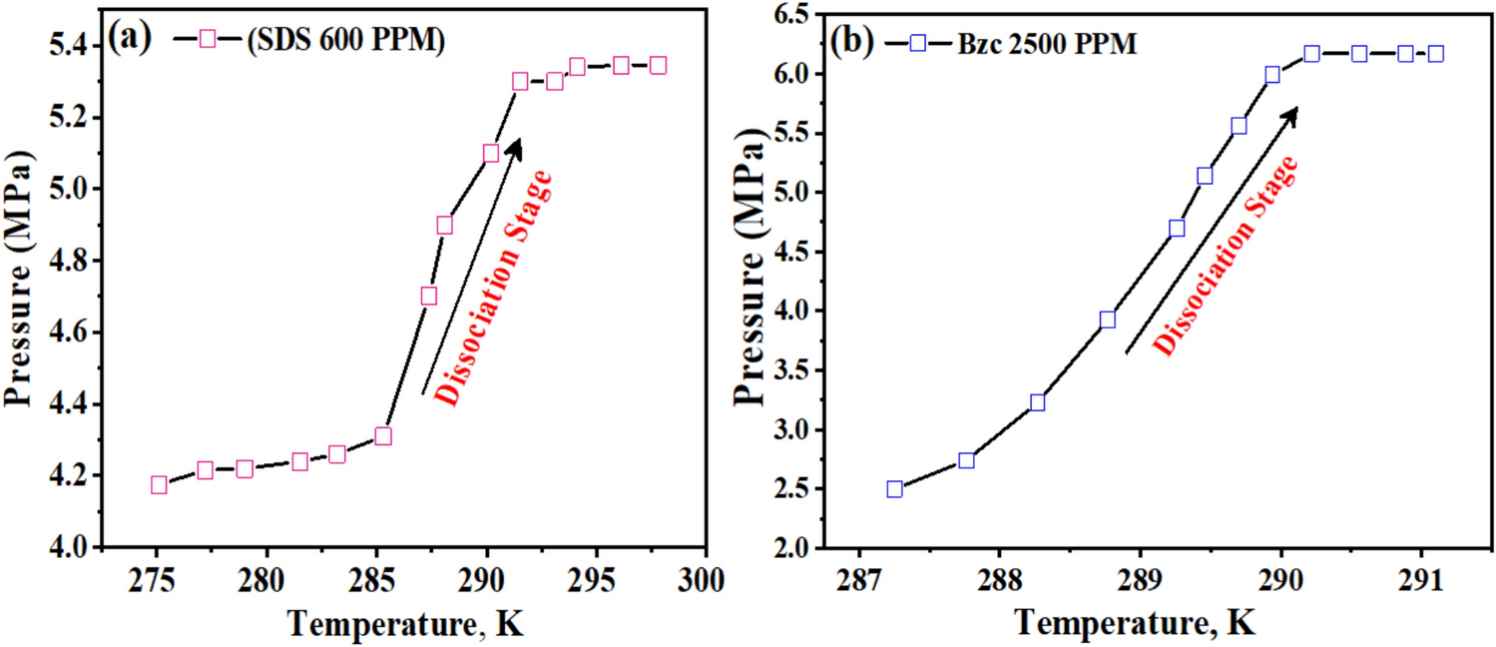
Pressure–Temperature plot during hydrate dissociation stage at **a** 600 ppm SDS Solution and **b** 2500 ppm Bzc.

The dissociation behavior of NGHs was investigated in the presence of two common additives, (SDS) and (Bzc), through pressure–temperature (P–T) measurements^1^. The SDS-containing system exhibited a distinct change in the slope of the dissociation curve in Fig. 12, suggesting a potential phase transition or structural alteration within the hydrate structure^59,60^.

In contrast, the P–T profile of the Bzc–NGH system showed a smoother, more continuous dissociation characteristic. These differences in dissociation patterns can be attributed to the interactions between the guest gas molecules and water molecules and the respective additive molecules within the hydrate lattice^61^. The findings contribute to understanding how the presence of different additives can significantly influence the stability and dissociation properties of NG hydrates. This is crucial for optimizing gas storage, transportation, and recovery processes and developing efficient hydrate management strategies.

### Mechanism of NGH formations

Natural gas consumption within the hydrate structure relies on the adsorption process between the clathrate (micelles and hydrate structure), which acts as the adsorbent, and NG molecules, which act as the adsorbate. This process can occur through one of the following cases: 1) Hydrogen bonding forms bonds between the absorbent and the adsorbate. 2) Adsorption by electron polarization occurs when the electron-rich sites on the adsorbent’s aromatic nuclei attract the positive sites of hydrogen atoms on the adsorbate (NG). 3) Adsorption by dispersion forces involves London-Van der Waals forces, acting between adsorbent and adsorbate, which are stronger with higher molecular weight adsorbents among the adsorbate (NG). Hydrophobic bonding occurs after the initiation of the formation of hydrates, and the first stage of NG adsorption is carried out (named as solid phase).

**Fig. 13:**
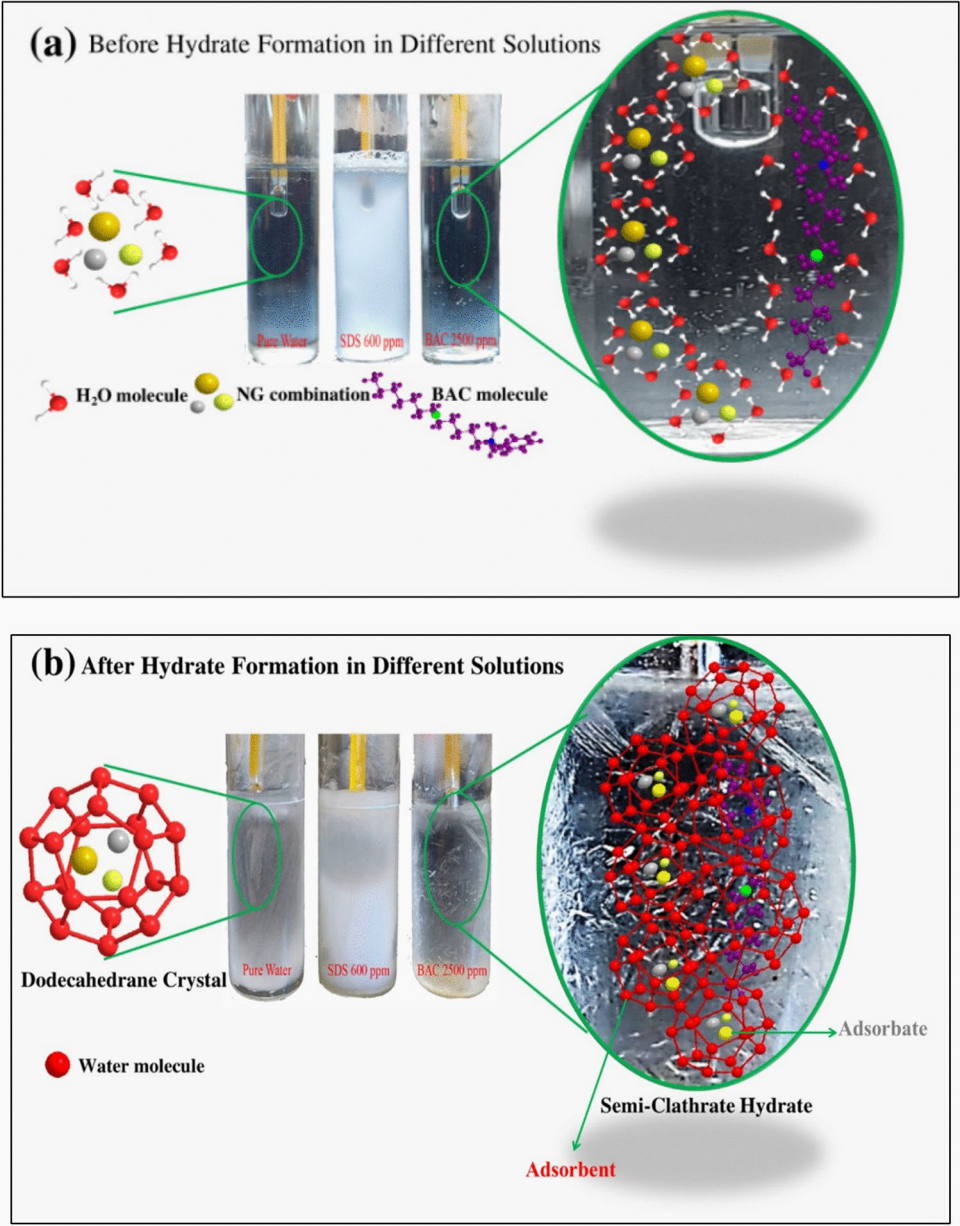
Mechanism of NGH before and after hydrate formation.

The continuous adsorption between the NG and the accumulating NG in the clathrate may be occurring by the hydrophobic bonding to continue the process. The mechanism of NGH formation, as illustrated in Fig. 13, demonstrates the influence of surfactants like SDS (600 ppm) and Bzc (2500 ppm) on hydrate stability and nucleation. Before hydrate formation, natural gas molecules are enveloped by a layer of water molecules, with surface-active agents strategically located at the boundary between the gas and liquid phases, thereby enabling molecular interactions. Post-hydrate formation reveals a distinct structural organization where water molecules form a pentagonal dodecahedral lattice (5^12^) around the gas molecules, particularly in the presence of surfactants^35^. This results in semi-clathrate hydrates, enhancing NGH stability and reducing induction time compared to pure water systems. The surfactants improve gas incorporation and hydrate formation efficiency by promoting nucleation at lower energy thresholds, which is critical for NG storage and transportation applications. This work represents an early-stage investigation focused on using Bzc to solidify natural gas in hydrate form. Compared to the NGH formation system with pure water, the new promoter (Bzc) eased the thermodynamic conditions required for NGH formation and significantly enhanced the formation kinetics and gas uptake. The importance of this research lies in exploring new potential semi-clathrate hydrates to improve NGH storage and developing practical approaches for implementing NGH formation under optimal conditions. After analyzing the results obtained from the measurements of electrical conductivity and zeta potential for surfactant SDS and Bzc, it became clear that the zeta potential signal, which indicates the stability of the electrical double layer of the solutions of the two surfactants, was higher for the Bzc than for SDS. Additionally, the electric charge on the surface-active group of the surfactant molecule was greater in Bzc than in the SDS. Primarily, this is done when focusing on the critical micelle concentration of both surfactants. This indicates that the Bzc is more stable in its solutions than the SDS, especially at the critical micelle concentration.

**Fig. 14:**
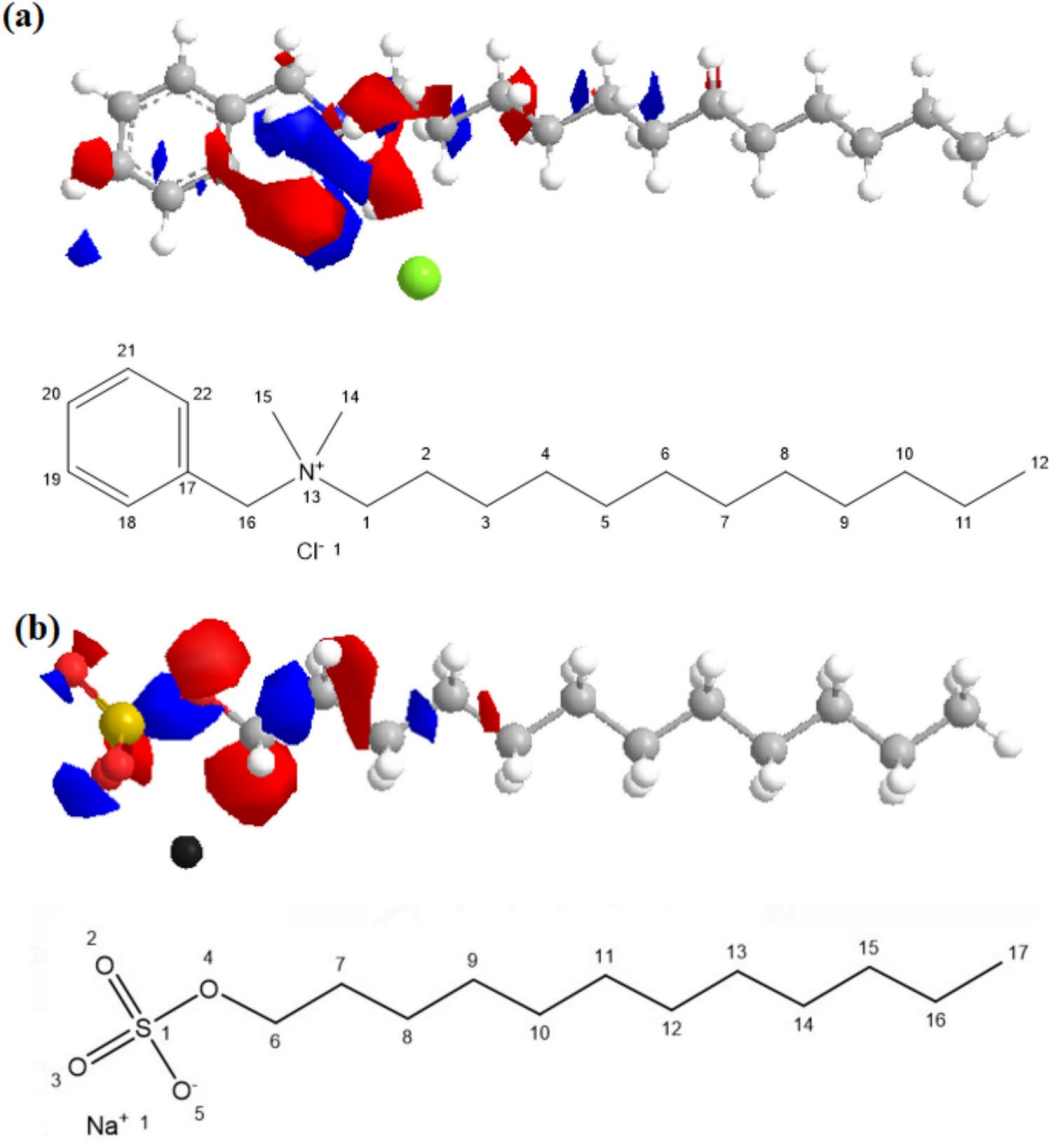
Chemical Structure and Electronic distribution charge for **a** Benzalkonium Chloride (Bzc) and **b** Sodium dodecyl sulphate (SDS).

The positive and negative charges are calculated according to the extended Huckel Theory, and their values are listed in Table 5. The obtained data showed that the net negative charge value, which is with Bzc, was − 1.711 eV. Meanwhile, the net charge value of SDS was -0.71; this finding explained why Bzc has more ability to hydrate formation, minimize the induction time, and maximize NG recovery among the SDS. The electronic charge distribution over the Bzc and SDS structure and the atoms within two compounds were numbered IUPAC system as shown in Fig. 14. The distortion in the chemical structure does not accurately reflect its spatial framework configuration. Instead, this distortion arises from the charge distribution across the molecular bonds. Specifically, it indicates the type of charge—negative or positive—dominating various atoms within the Bzc molecule, as according to the extended Hückel theory, where red and blue colors represent partial negative and positive charges, respectively.

**Table 5:** Charge distribution for Bzc and SDS according to extended Huckel theory.

No	Bzc		SDS	
	Atom number	Charge value (eV)	Atom number	Charge value (eV)
1	C (1)	0.009689	C (6)	0.14863
2	C (2)	− 0.05977	C (7)	− 0.0704
3	C (3)	− 0.05765	C (8)	− 0.05113
4	C (4)	− 0.05561	C (9)	− 0.05566
5	C (5)	− 0.05555	C (10)	− 0.05547
6	C (6)	− 0.05553	C (11)	− 0.05553
7	C (7)	− 0.05553	C (12)	− 0.05552
8	C (8)	− 0.05549	C (13)	− 0.05549
9	C (9)	− 0.05535	C (14)	− 0.05534
10	C (10)	− 0.05383	C (15)	− 0.05383
11	C (11)	− 0.04653	C (16)	− 0.04653
12	C (12)	− 0.1288	C (17)	− 0.1288
13	N (13)	0.745899	Na (1)	0.99999
14	C (14)	− 0.10937	S (1)	2.7534
15	C (15)	− 0.05156	O (2)	− 1.1104
16	C (16)	0.022884	O (3)	− 1.10986
17	C (17)	0.06898	O (4)	− 0.44474
18	C (18)	− 0.07845	–	–
19	C (19)	− 0.0443	–	–
20	C (20)	− 0.05341	–	–
21	C (21)	− 0.03933	–	–
22	C (22)	− 0.06354	–	–
23	Cl (23)	− 1	–	–
Total positive charge		0.847452 eV	3.90202 eV	
Total negative charge		− 2.1196 eV	− 4.60351 eV	
Total net charge		− 1.7211 eV	− 0.70149 eV

These electronic and physical properties placed the Bzc at the forefront when it was chosen to form NGHs. It worked to store it more effectively than the SDS, commonly used in this technological field, as shown in Tables 3, 4, and 5 and Figs. 1, 2, 3, 4, 5, 6, 7, 8, 9, 10, 11, 12, 13, and 14, the Bzc achieved the best results regarding the induction time taken to form hydrates, phase equilibrium stability, and the temperature and pressure used for its formation, addressing some shortcomings in using the SDS. This confirms that the wisdom of selecting Bzc as the primary material for investigating the kinetics of formation, induction time, and the optimal pressure required for hydrate formation, as well as the process of hydrate dissociation and comparing it with a commonly used material, was successful in addressing the shortcomings in the performance of material SDS.

## Conclusions

The experimental results from this study emphasize the significant impact of (Bzc) on the thermodynamic and kinetic properties of NGH. Phase equilibrium analysis reveals that while SDS doesn’t significantly affect the pressure and temperature conditions for hydrate formation, the introduction of Bzc markedly reduces the hydrate formation pressure to 2.4 MPa whereas (up to 4.6 MPa for SDS) and shifts the nucleation’s temperature by 7.5 K compared to a pure water system. These findings suggest that Bzc could be a highly effective promoter for natural gas storage in hydrates, potentially lowering operating costs due to reduced formation pressures. The kinetic analysis further demonstrates Bzc’s effectiveness as a promoter, evidenced by shortening the induction time and increasing the cumulative gas consumption, improving the hydrate formation rates, and enhancing the storage capacities. Specifically, the supplement of Bzc decreases the induction time of hydrate formation to 9.9 min (41.3 min. for SDS), significantly accelerating the overall hydrate formation process from nucleation to complete growth. Additionally, Bzc ensures faster and more efficient gas recovery during hydrate dissociation, with NG recovery reaching 98.56% for Bzc hydrate formation and 95.38% for SDS hydrate formation, albeit in a short time with Bzc. This concluded that the Bzc emerges as a promising promoter for NGH storage, offering benefits such as reduced formation pressures, quicker formation rates, shorter induction times, and greater storage capacities than the SDS.

## Supplementary Information

Supplementary Information.

## Data Availability

The data that support the findings of this study are available from the corresponding author upon reasonable request. Email: Eng_msalah1@yahoo.com.
