# Supplementary material for: Innovative role of Benzalkonium chloride as a quaternary ammonium salt for natural gas hydrate formation and storage
Source: Sci Rep. 2025 May 9;15:16144. doi: 10.1038/s41598-025-97520-3 (PMC12062228; doi:10.1038/s41598-025-97520-3)
Supplement: Supplementary file 1 — Supplementary Information. [file 41598_2025_97520_MOESM1_ESM.docx]

## **Highlight:**

## NG storage via forming NG/Bzc hydrate system was investigated.

## Bzc significantly strengthened the hydrate formation kinetics.

- Bzc at 2500 ppm reduced hydrate nucleation induction time to 9.9 minutes, compared to 41.3 minutes with SDS.
- Significant improvement of nearly **76%** in induction time reduction.
- NG/Bzc hydrate showed a good storage capacity.
- Notable enhancement about 98.65% of NG recovery after dissociation stage.
